# ‘Research is the last thing on our minds, we are in crisis’: Experiences of Lagos state nurses towards research and scholarly endeavours

**DOI:** 10.1002/nop2.1967

**Published:** 2023-08-15

**Authors:** Oluwadamilare Akingbade, Oluwadara Eniola, Adesina A. Sulaiman, Emmanuel O. Adesuyi, Esther B. Ilesanmi, Zainab O. Adesokan, Bose C. Ogunlowo, Rafiat T. Akinokun, Emmanuel Oviri, Chioma J. Eze, Bolanle O. Ayodele

**Affiliations:** ^1^ Institute of Nursing Research Osogbo Nigeria; ^2^ The Nethersole School of Nursing The Chinese University of Hong Kong; ^3^ Department of Nursing Birmingham City University Birmingham UK; ^4^ Directorate of Nursing Ministry of Health Ikeja Lagos State Nigeria

**Keywords:** experiences, Nigeria, nurses, qualitative study, research, research capacity

## Abstract

**Aim:**

To explore the experiences of Nigerian nurses in research and scholarly endeavours.

**Design:**

Descriptive phenomenological qualitative study design.

**Method:**

In‐depth interviews were conducted among 30 nurses until data saturation was reached. Data were analysed using the thematic analysis method, and consolidated criteria for reporting qualitative research guidelines (COREQ) were adhered to in reporting this study.

**Results:**

Three themes emerged: (1) challenges of nurses with research; (2) the state of nursing research in Lagos state; (3) strategies to improve the state of nursing research in Lagos state. Some challenges nurses in Lagos State encounter with research include heavy workload and nursing shortage due to brain drain, faulty research foundation, lack of continuing education in research and the stressful nature of conducting research in Lagos.

**Conclusion:**

As research is essential in addressing healthcare challenges, policymakers are encouraged to employ more nurses to reduce the workload and provide time for research activities. Training and continuing education in research can be incorporated into professional development programmes. Lagos State will benefit from nursing research units in the hospitals, and a coordinating centre for the units should be provided with adequate funding. Support could be obtained from the Institute of Nursing Research Nigeria in establishing these structures. Hospital management should embrace and welcome research output from nurses and ensure such results are implemented to improve patients' care. Nurses who excel in research might be recognised with awards and other incentives to inspire their peers.

## 1 INTRODUCTION

Healthcare delivery is becoming more dynamic with various health challenges surfacing, requiring healthcare practitioners and nurses to think critically, systematically analyse situations and make clinically sound decisions (Li et al., 2019). Globally, the research output of nurses has increased over two decades, with developed countries taking the lead and a disproportionate output from low‐ and middle‐income countries (Yanbing et al., 2021). Critical gaps have been observed in nursing and midwifery research in Africa with poor skills and research output, which can be attributed to inadequate funding, lack of job satisfaction, poor access to technology, ineffective interprofessional education and practice and few PhD‐prepared nurses (Klopper & Gasanganwa, 2015).

Research capacity in nursing has been defined as ‘the ability to conduct nursing research activities sustainably in a specific context’ (Chen et al., 2019). Ayandiran et al. (2013) observed that various modifications had been made to the structure of nursing education and practice to improve nursing research capacity in Nigeria. For instance, nursing education was upgraded from hospital‐based training to higher education institutions with a better focus on developing scientific knowledge and research. However, the nursing research capacity in Nigeria is not yet optimal (Ndubuisi et al., 2021).

According to Udoye (2018), postgraduate degree holders are key to determining the quantity and quality of research output in a field. This is because they are better equipped with the knowledge and skills to effectively conduct and disseminate research findings (Klopper & Gasanganwa, 2015). Even while postgraduate degrees, particularly PhDs, are expected to be the source of higher‐quality research output in a field, there is a limited number of nurses with these higher degrees in Nigeria. A report revealed that between 1988 and 2014, out of the 880 postgraduate nursing students (MSc and/or PhD) admitted to Nigerian universities, only 88 students could complete the degrees. Similarly, there is a paucity of postgraduate nursing programmes in Nigeria (Onwe, 2018). The state of postgraduate nursing education in Nigeria is expected to influence research output from the country.

According to the study conducted by Oluwatosin (2014) in Nigeria, only 21.2% of nurses had conducted clinical research based on their experience, while 4.4% had published in a peer‐reviewed journal out of a sample of 325 nurses. This shows that nurses' research output is critically low. Similarly, Emenloye et al. (2016) reported that although 25% of nurses in Nigeria have bachelor's degrees, they do not possess the necessary knowledge to conduct nursing research.

Some challenges facing nursing research in Nigeria have been documented. They include excessive workload, inadequate staffing, lack of nurse researchers, cost, lack of mentors in nursing research, lack of guidance with disseminating research findings, poor internet service and lack of organisational support (Edet et al., 2011). Similarly, Oluwatosin (2014) observed the lack of capacity‐building programs and facilities in most organisations' nursing units or departments (Oluwatosin, 2014). Furthermore, the Federal Ministry of Health (FMOH) in Nigeria (2015) reported a meagre allocation of funds to research (about 0.08%), instead of the usual 2%, with most of the funds used at the federal level, leaving nothing for the States.

To the best of our knowledge, there is a paucity of recent literature focused on the experiences of Nigerian nurses in research and publications, which prompted this study to explore the experiences of Nigerian nurses in research and scholarly endeavours.

## 2 METHODS

### 2.1 Study design

As the study focused on exploring the lived experiences of Lagos State nurses regarding research, a descriptive phenomenological qualitative study design (Shorey & Ng, 2022) was adopted. The consolidated criteria for reporting qualitative research guidelines (COREQ) were adhered to in reporting this study. See Supplementary material.

### 2.2 Participants and settings

The study was conducted among nurses from selected healthcare institutions in Lagos State, Nigeria. Participants were selected using a purposive sampling technique considering various ranks, years of experience and facility (See Table 1). The study was conducted in Lagos State, located in the South‐Western part of Nigeria. Lagos State is the smallest in Nigeria in terms of size but has the highest urban population, which was 24.6 million inhabitants as of 2015. Lagos has a high mix of tertiary, specialist, general and private hospitals (Lagos State Government, 2022).

**TABLE 1:** Sociodemographic details of the participants.

Variable	Frequency	Percent
Mean Age 37.5 ± 10.16		
Gender		
Male	7	23.33
Female	23	76.67
Educational Level		
RN\Post‐Basic	6	20.00
Bachelor's Degree	18	60.00
MSc	4	13.33
Masters in view	1	3.33
PhD in View	1	3.33
Hospital Setting		
Secondary	10	33.33
Tertiary	17	56.67
Ministry of Health	2	6.67
National Association of Nigeria Nurses and Midwives (NANNM)	1	3.33
Rank		
Nurse‐Intern	1	3.33
Nursing Officer II (NOII)	4	13.33
Nursing Officer I (NOI)	7	23.33
Senior Nursing Officer (SNO)	6	20.00
Principal Nursing Officer (PNO)	3	10.00
Assistant Chief Nursing Officer (ACNO)	3	10.00
Assistant Director of Nursing Services (ADNS)	4	13.33
Deputy Director of Nursing Services (DDNS)	1	3.33
Director of Nursing Services (DNS)	1	3.33
Years of Experience		
Less than 5 years	8	26.67
6–10 years	7	23.33
11–15 years	5	16.67
16‐20 years	4	13.33
21‐25 years	3	10.00
26 and above	3	10.00
Years of Experience in Research		
Less than 5 years	25	83.33
6–10 years	2	6.67
11–15 years	2	6.67
16 and above	1	3.33
How much research have you conducted?		
Less than 5	28	93.33
6–10	2	6.67
Number of publications		
Nil	25	83.33
1 publication	3	10.00
2 publications	2	6.67
Presentation in a local or international conference		
Nil	27	90.00
1 presentation	3	10.00

### 2.3 Data collection

Individual in‐depth interviews were conducted among 30 nurses from February 2022 to April 2022 until data saturation was reached. Data saturation refers to a state in which new insights or meanings can no longer be drawn from the data collected (Glaser & Strauss, 1967). Each interview took about 30–40 min. The study included all cadres of registered nurses employed and working in any healthcare facility in Lagos State, Nigeria. However, retired nurses and nurses employed in states other than Lagos were excluded. The interviews were conducted online via Zoom. The first and second authors, male registered nurses with PhD and bachelor's degrees in nursing with experience in qualitative data collection, conducted the interviews. An information sheet with the research details was presented to the participants, and their consent was obtained before the interview, after which demographic data were collected. Experts in nursing research developed the semi‐structured interview guide from a detailed literature review. The interviews were conducted virtually and were audio‐recorded after the participants gave their consent. The structure of nursing cadre in Nigeria is such that years of experience is a major determinant for getting promoted to another cadre. A minimum of 3 years of experience is required for promotion from one nursing officer cadre to the next.

### 2.4 Data analysis

**FIGURE 1:**
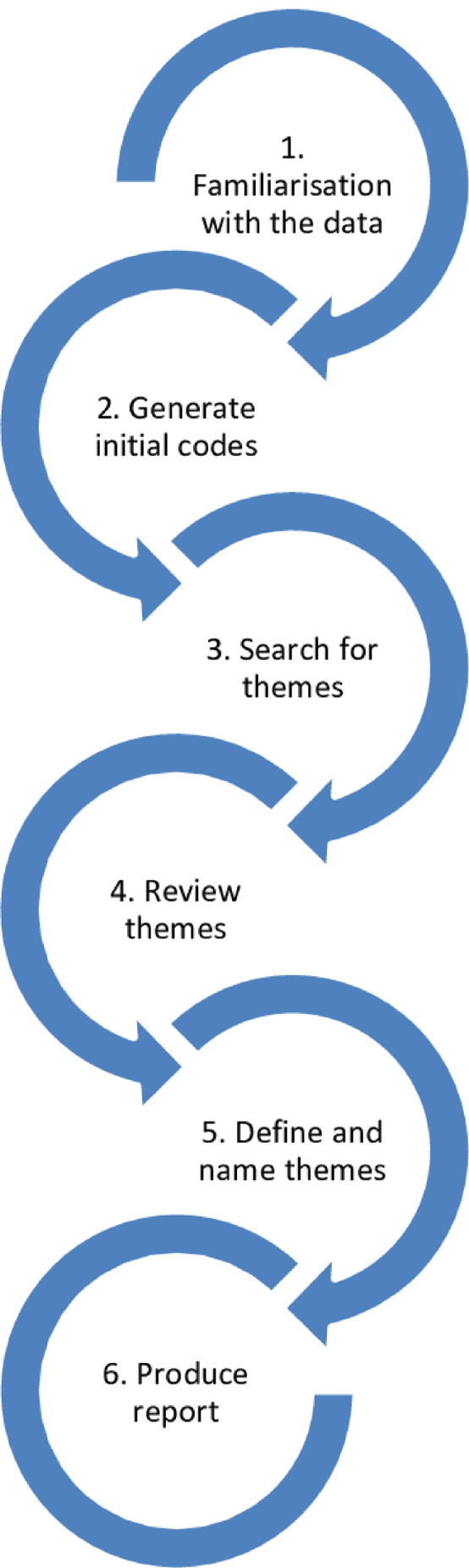
Six‐Step Approach to Thematic Analysis.

Data were analysed using the NVIVO 12 software. The authors trained in data transcription did a verbatim transcription of the recordings. Thematic analysis was utilised for data analysis. The six phases include: familiarising with the data, generating initial codes, searching for themes, reviewing themes, defining and naming the themes, and producing the report (Braun & Clarke, 2019; Polit & Beck, 2019). See Figure 1.

Various strategies were taken to ensure rigour in this study. Credibility and confirmability were ensured through member checking and peer debriefing, while dependability and transferability were ensured through audit trails and detailed method descriptions (Anney, 2014; Elo et al., 2014).

### 2.5 Ethical consideration

Ethical approval to conduct the study was obtained from the Ethical Review Committee (ERC) of the Lagos University Teaching Hospital (LUTH) and Lagos State Health Service Commission (LSHSC), with approval numbers: ADM/DSCST/HREC/APP/4930 and LSHSC/DNS/RESEARCH/VOL III/169 respectively. In addition, informed consent was gained from participants before joining the study. Confidentiality was assured, and the participants' right to refuse to participate was respected.

## 3 RESULTS

Table 1 shows that most of the respondents were females (76.67%) with bachelor's degrees (60%) working in tertiary settings (56.67%). The respondents were drawn from the various ranks in nursing, with Nursing Officer I having the highest percentage (23.33%). The years of experience also varied among the participants, with 26.67% having less than 5 years of experience in nursing. Although 73.33% had more than 5 years' experience in nursing, 83.33% had less than 5 years' experience in research, and 93.33% had conducted less than five research. More than 80.00% do not have any publication, and 90.00% had not presented a paper at a local or international conference.

Three major themes emerged: challenges of nurses with research, the state of nursing research in Lagos State, and strategies to improve the state of research in Lagos State. The themes were elaborated into various sub‐themes (See Table 2).

**TABLE 2:** Themes and Sub‐themes.

Themes	Sub‐themes
Challenges of nurses with research	The challenging nature of the clinical environment Faulty foundation in research Lack of continuing education in research The stressful nature of conducting research in Lagos Poor institutional support Theory‐practice gap in nursing
The state of nursing research in Lagos state	The attitude of nurses to research Little research is done by nurses Poor research funding Perceived competence of nurses in research Research needs of nurses and research capacity of nursing departments
Strategies to improve the state of nursing research in Lagos State	Improved awareness in research Increase funding for research Need for research centre, research units and research nurses Continuing education in research Support from institutions, governmental and non‐governmental organisations Involve young and male nurses in research Train the trainers

### 3.1 Theme 1: Challenges of nurses with research

Six categories of the participants' concerns regarding their challenges with research came to the fore. They are further elaborated under the following sub‐themes:

#### 3.1.1 The challenging clinical environment

The participants verbalised their concerns about the demanding nature of the clinical environment. They recounted the challenges they encountered while rendering their services, including a heavy workload, a shortage of nurses and limited time available for research.

When you have only an intern‐nurse and maybe a staff nurse to run a 30‐bed ward, research is the last thing on our minds. It's tough; we are in crisis. (P15, 55 years, ADNS)

In Lagos state, we don't have the time of our own, and the shortage of nurses is killing everybody. I have worked round the clock since yesterday till this moment, and somebody is telling me that I have to carry out research, it will not work. (P10, 55 years ADNS)

…if someone is running a 16‐hour or 24‐hour shift with no free time, with family to cater for, combining this will not be possible. (P4, 35 years, SNO)

#### 3.1.2 Faulty foundation in research

Some participants decried the quality of research they were exposed to as students. Some lamented that they did not have good supervisors, while others were not interested in research.

I realised that the supervisors are practically the problem of the students. Some don't know how to simplify supervision; they make it so complex. Because they have the privilege to supervise your project, they take advantage of that to rubbish your work without giving you directions. Research supervisors are supposed to be like mentors, but they often condemn whatever you do without giving proper guidance. (P20, 40 years, ACNO)

…the research I did in the nursing school then was not top‐notch; it truly affected my interest in it. Although I knew it was necessary, I did not fancy it. I saw it as an academic requirement to move to the next level. (P25, 30 years, NO II)

We did not take it seriously because of how we were taught. So, my competency is so low; honestly, I cannot regard myself as a researcher. (P22, 40 years, ACNO)

#### 3.1.3 Lack of continuing education in research

Many participants also revealed that they had not been exposed to continuing education in research since they graduated from their schools.

…after school, there's no training on research, most of the training we do is just to renew our license and keep the work going‐ the MCPDP or something…. (P18, 35 years, NO I)

#### 3.1.4 The stressful nature of conducting nursing research in Lagos

A major concern of the participants was the stress associated with conducting research in Lagos state.

Conducting research in this Lagos state is a bit stressful. Like the last I did on children with neuro disorders, sometimes, before I get there, it's already rowdy; mothers are not patient. If I give out ten questionnaires, hardly will I get five back because everybody is concerned about seeing their doctors. Even after that, they just want to go home. Even for me, moving from Ikorodu to Lagos Island to go and get data is very stressful. (P18, 35 years, NO I)

#### 3.1.5 Brain drain

Another major challenge that emerged was the loss of nurses that are good with research through migration for greener pastures.

…those nurses I know that are excellent in research are leaving the country for better jobs, and once they go, they go with all their intellectual abilities. (P8,39 years, PNO)

#### 3.1.6 Poor institutional support

The participants lamented their institutions' poor support in academics and research.

And another thing is that the system does not encourage you like you want to do research okay o, let's give her two weeks break because she's doing research, nooo, you will do it on your normal duty, who sent you research (laughs). Even if they take you to the nursing leaders, they will tell you, who sent you, is it part of the work we employed you to do…. (P25–30 years NO II)

…even when you are trying to go to the academic area, that is, you want to go and do your masters, Lagos state will not support you to go and do masters unless you leave your work, they just want you to work and work. (P9, 30 years, SNO)

### 3.2 Theme 2: The state of research in Lagos state

This theme provided an insight into the current state of nursing research in the State, the attitude of nurses to research and the research needs of nurses. This theme is further discussed under five sub‐themes.

#### 3.2.1 The attitude of nurses to research

This sub‐theme reflects the various attitudes of nurses in the State to research.

…our attitude towards research is very poor. Since I left school, I have not conducted any research, and I have not seen any research conducted in my hospital that is really nursing‐related. (P26, 29 years, NOI)

Some participants lamented that they were not interested in research because there is a huge gap between research and practice.

I do not think the research done is being implemented. There is a massive gap between what we are finding and what is being done. (P16, 24 years, Nurse‐Intern)

While the majority opined that the attitude of nurses to research is poor, some observed that nurses have a good attitude to research.

I enjoy doing research anyway, I love trying to find solutions to problems. I think it's my personality. Even when I'm not appreciated, I enjoy doing research. (P28, 29 years, NOI)

I believe that Lagos state nurses are eager to do stuff. They are eager, my friends are eager. Even if you come to my ward, my matron is eager. (P7, 27 years, NO I)

#### 3.2.2 Minimal research by nurses

Some participants observed that nurses are lagging in research in the State. Similarly, many declared that they had no experience in publication of findings.

…most of the research is done by doctors, and the administration believes that doctors will do it better. They just believe that whatever we do is just by the way. (P26, 29 years, NO I)

Research publication, haa, no, although, I want to join some people; but presently, none. (P18 35 years NO I)

I don't have experience in publishing. You know Nigeria is not helping when you come to that…. (P30, 40 years, PNO)

Although many participants opined that nurses do little research and not many have a good attitude towards the research, some believed that younger nurses seem more versatile in research.

…the new generation of nurses are younger, more versatile and with better education. They seem to have a flair for research, but the way we were brought up, we were not brought up to be researching, so the little research was merely part of the requirement for the nursing council exam (P18, 51 years, PNO)

### 3.3 Poor funding for nursing research in the State

Many participants decried nurses' limited remuneration alongside the unavailability of grants for conducting research.

Nurses' income is meagre. There's hardly any grant to carry out any research. So, if anyone wants to do research, it would be from their purse, no grant no support. (P5, 54 years, DDNS)

…we are underpaid, so you can't imagine the little amount you're earning that you have to start pumping into the research, except you have grants. (P9, 30 years, SNO)

#### 3.3.1 Perceived competence of nurses in research

Majority of the participants rated their competence in research as average.

I will say 50%. If there is a need to conduct research, I will need to dust my books so I can get to like 60%, then maybe with proper guidance, I can get to 70. Who will (P8, 39 years, PNO)

…maybe five on a scale of 1‐10 (laughs). Due to my location, nobody is encouraging you; even when you have the knowledge, the environment will kill it. So maybe when I find myself in an environment that wants to use my findings, that will encourage me to do better. (P30, 40 years, PNO)

#### 3.3.2 Research needs of nurses and research capacity of nursing departments

Several research needs came to the fore during the interviews. While the majority claimed they needed training in all the areas of nursing research, there was a preponderance of needs in research methodology and data analysis.

I need training in all areas of research. If there is an online platform that nurses can connect to from the comfort of their homes, they can learn at their pace, this will be helpful. (P28, 29 years, NO I)

…I'll need to brush through the research methodology and data analysis all over again…. (P12, 31 years, SNO)

Many of the participants perceived that although the nursing departments have the capacity to conduct research, many of them are currently not doing that. Many reported that their hospitals do not have a research unit but an education unit. Few of them claimed their hospitals had a research unit.

Aah, well, for my institution, I can still say that the capacity is below average. You know we are just starting, but will there be coordination, will they have time to go about it…these are the questions. (P3, 54 years, SNO)

### 3.4 Theme 3: Strategies to improve the state of research in Lagos state.

Seven categories of strategies emerged from the interviews. They are further elaborated under the sub‐themes below:

#### 3.4.1 Increased awareness in research

Some participants observed that nurses would have been more involved in research if they had been more enlightened.

…When you are enlightened, you don't do things the old way. The awareness should not just be for the young ones, start from the top, capturing the young ones is not a problem. (P28, 29 years, NO I)

#### 3.4.2 Increased funding for research

The participants opined that many nurses would be involved in nursing research is funded.

It takes a lot because they need money to do many things. If nurses can get sponsorship to carry out their research, I think many people will try to get involved. (P5 55 years ADNS)
.

#### 3.4.3 Need for research Centre, research units and research nurses

The participants observed that if relevant structures were put in place, nursing research would be successful in the State. Some structures include a coordinating research centre, hospital research units, and research nurses focused solely on research.

Yes, it will be great if we have a research centre in Lagos. We can utilise the Lagos State research sub‐committee of the NMCN to reach out to all institutions in the state. So, if we have a consultant that can work with us to ensure that the subcommittee works, we are ready to go. (P1, 55 years, DNS)

Having a research unit in the hospital is another good idea, where nurses can work as researchers. Because many of the nurses at the bedside have these things in them, but they are dying. (P2, 35 years, NO I)

…we can have nurses that their work is basically research, and they are in that research unit. it is possible, it happens in other countries, so I don't think ours should be an exemption. (P29 45 years, SNO)

#### 3.4.4 Continuing education in research

As some of the participants reported that they had not been involved in research training since they graduated from their schools, the place of continuing education in research was stressed.

I admonish that it should be embedded in our unit clinical training settings for continuous education; this will help. (P5 54 years ADNS)

#### 3.4.5 Support from institutions, governmental and non‐governmental organisations

The participants also emphasised the need for more support from the relevant stakeholders in the State.

…if Lagos state can do it in a way that upon approval, researchers will be given a time frame to conduct their research with job security. (P4, 35 years, SNO)

On the part of the government, they should try to make this country a research place. They should play their part by funding nursing research. Some NGOs will always want to support research but fall back if the government does not align. (P6, 40 years, PNO)

#### 3.4.6 Train the trainers

While some of the participants lamented their experiences with research supervision, some recommended the need for training courses for research supervisors.

The people who are supervising research should be trained. There is something called “train the trainer” The trainers should be trained properly; the people who are supervising research are not helpful. (P20 40 years, ACNO)

#### 3.4.7 Involve young and male nurses in research

Some participants observed that male nurses seem more interested in research and can mobilise the younger nurses; hence, they recommended that they be involved in research.

Male nurses are learned, and I think they are interested in research. If they can be approached because they influence the junior nurses, I think they will know how to mobilise others. (P25 30 years NO II)

## 4 DISCUSSION

Clinical decision‐making is based on sound, evident, valid and currently available research output which helps in optimal healthcare (Coyne et al., 2016). Nurses are open to various roles in research ranging from being a participant or investigator to an end user, i.e. utilising the outcomes (Hernon & Dalton, 2019). However, being an investigator and also utilising outcomes require knowledge and experience in research. This study aimed to explore the experiences of Lagos State nurses in research. The first theme in this study highlighted the challenges nurses face with research at the individual and institutional levels. Nurses in Lagos State were limited by various barriers arising from their work environments, such as heavy workload and shortage of nurses. Similar to the findings of a recent qualitative research study in Australia by Hines et al. (2022) where several organisational issues surrounding work cultures, staffing level, disinterest and supportiveness were emphasised by nurses as challenges facing their fitting into their sphere of research in their clinical nursing practice. This also conforms to a previous finding by Abam et al. (2014) among nurses in Calabar, South–South Nigeria, where excessive workload on the ward and inadequate staffing was a major constraint in conducting research. Earley (2014) identified that research methodology and the utilisation of research outputs in real‐life situations are mostly taught in tertiary institutions, however, nurses in Lagos State recounted their faulty foundations in research and lack of continuing education in research, which may have an impact on their interest in conducting research as a graduate nurse. This conforms to the study of Wu et al. (2019) which revealed that research education and training are essential to improving the interest and skills of nurses in conducting research.

Another major challenge pointed out by this study was the poor institutional support nurses that nurses receive. Lagos is a diverse and overpopulated state, with many clinical responsibilities placed on nurses. Unfortunately, due to short‐staffing, hospitals do not approve leave or break for workers to participate in research activities, which further reduces nurses' interest in carrying out research. This challenge aligns with the finding of Asuquo et al. (2013), which indicated that most nurses are not well represented at the institutional level in decisions related to research. Furthermore, the Nigerian health sector is currently witnessing the “brain drain” phenomenon, wherein the country's best professionals are leaving the country for academic or professional purposes (Lawal et al., 2022). This has affected all aspects of healthcare delivery, including research activities (Olorunfemi et al., 2020). This study revealed that the migration of nurses out of the country is a challenge affecting research capacity as many that are good at research are leaving the country's shores, taking along all their intellectual abilities.

The second theme reflected the state of nursing research in Lagos state. Findings from this study showed that although research is mandatory for awarding a nursing certificate from diploma to postgraduate level (Emelonye et al., 2016), most nurses do not get involved in clinical research after graduation, re‐iterating the poor attitude of nurses to research. This is consistent with the study carried out by Abam et al. (2014), where nurses were found to have a low motivation towards research due to a lack of enabling environment and support. There is, therefore, a need to motivate nurses to see research as an integral part of their practice while also providing support and encouragement to carry out research. Perceived research competence among nurses in this study was placed at an average. Although a study by Grande et al. (2021) showed that most nurses are familiar with the research process. However, training programmes for nurses are crucial to developing competence in research (Wu et al., 2019).

The third theme for this study was the strategies to improve the state of research in Lagos state. Enlightenment of nurses on the importance of research was one of the major strategies. Furthermore, as the need to improve funding of nursing education in Nigeria has been identified in the literature (Arowolo et al., 2023), adequate funding to cover research expenses will go a long way in increasing the research capacity of nurses (Torres et al., 2017). In addition, the Federal Ministry of Health (FMoH), in Nigeria has reported low annual budget funds for research in the country (FMoH, 2015). An upward review of the budget with a focus on nursing research is hereby warranted. This can also help in accessing high‐quality studies published in open‐access journals. These have improved the research capacity of nurses in Scotland (Hu et al., 2019).

Although there are professional continuing development programmes for nurses, many of our respondents submitted that research is missing from the content of these programmes and that nurses merely attend such programmes for the purpose of licence renewal. Hence, we recommend a review of these programmes with research content. Also, as the use of digital devices increased in Nigeria during the COVID‐19 pandemic (Akingbade et al., 2022; Akingbade et al., 2023), and recent research revealed that Nigerian nurses find virtual learning impactful; continuing education programmes can also leverage online platforms to build research competencies of nurses, as is the case at the Institute of Nursing Research Nigeria (Adesuyi et al., 2023). However, it will be important to address the challenges that some might encounter with internet connectivity (Ogbeide et al., 2022).

Furthermore, as Ndubisi et al. (2021) identified that there is a limited number of nurses in the system who can provide mentoring and supervision to young researchers; therefore, it is essential to train nurse leaders and lecturers to ensure they provide adequate training with a focus on young nurses as reported in this study. Furthermore, as postgraduate programmes play a crucial role in the preparation of nurses with competence in research, we recommend the provision of more funds to support postgraduate programs in Nigeria, as evidence suggests that these programmes are few and underfunded (Onwe, 2018). Owing to barriers such as lack of time and unavailability of funds, support from concerned stakeholders is one of the strategies for improving the state of research. This is consistent with the findings of previous studies that identified leadership as one of the factors influencing the involvement of nurses in research (Li et al., 2019; Warren et al., 2016).

Additionally, as our study participants recommended the establishment of a research unit in hospitals managed by research nurses, and a centre for the coordination of such activities, we recommend collaboration with the Institute of Nursing Research (INR) Nigeria for technical support in establishing these units and centres. Research‐dedicated nurses and units in each hospital will be beneficial in improving the competencies of clinical nurses in research (Mulkey, 2021). Similarly, we recommend further collaboration between academic and clinical institutions on research projects. Furthermore, support can be obtained from INR Nigeria for the training of the nurses in research to improve their research knowledge and capacity. Similarly, as funding is crucial for the establishment of such initiatives, we recommend financial support from the Lagos State Ministry of Health and other local and international organisations to make this project a reality.

### 4.1 Implication for policy

To improve the research knowledge and skills of Lagos state nurses, policymakers in nursing should incorporate training and continuing education in research into the professional development programmes. Also, as time is a significant constraint to conducting research, nurses can be given a break or leave for research activities. Hospital management should embrace and welcome research output from nurses and ensure such results are implemented to improve patients' care. Lastly, awards and other means of motivation can be used to encourage research participation.

### 4.2 Strength and limitation

As this descriptive phenomenological qualitative study was conducted among a few nurses in Lagos, Nigeria, this might not reflect the experiences of nurses in other States in Nigeria, which might limit the generalisability of the study. Hence, this should be considered when interpreting the findings. However, this study has addressed a significant gap in the literature.

## 5 CONCLUSION

This study has provided insight into the experiences of Lagos State nurses in research alongside the challenges they face and strategies to improve their research capacity and that of nursing departments in the state. It is hoped that suggestions from this study will improve the state of nursing research in Lagos state.

## AUTHOR CONTRIBUTIONS

Study design: OA, OE, AAS, EOA, EBI, ZOA, BCO, RTA, EO, KA. Data collection: OA, OE, AAS, EOA, EBI. Data analysis: OA, CJE. Manuscript writing: OA, OE, AAS, EOA, EBI, ZOA, CJE. Critical revision: OA, OE, AAS, KA. Study supervision: OA, OE, KA.

## FUNDING INFORMATION

No funding was secured for this study.

## CONFLICT OF INTEREST STATEMENT

The authors do not have any potential conflicts of interest to declare.

## Supporting information


Appendix S1.

## Data Availability

The data that support the findings of this study are available from the corresponding author upon reasonable request.
